# Human attention affects facial expressions in domestic dogs

**DOI:** 10.1038/s41598-017-12781-x

**Published:** 2017-10-19

**Authors:** Juliane Kaminski, Jennifer Hynds, Paul Morris, Bridget M. Waller

**Affiliations:** University of Portsmouth King Henry 1st Street, Portsmouth, PO12DY UK

## Abstract

Most mammalian species produce facial expressions. Historically, animal facial expressions have been considered inflexible and involuntary displays of emotional states rather than active attempts to communicate with others. In the current study, we aimed to test whether domestic dog facial expressions are subject to audience effects and/ or changes in response to an arousing stimulus (e.g. food) alone. We presented dogs with an experimental situation in which a human demonstrator was either attending to them or turned away, and varied whether she presented food or not. Dogs produced significantly more facial movements when the human was attentive than when she was not. The food, however, as a non-social but arousing stimulus, did not affect the dogs’ behaviour. The current study is therefore evidence that dogs are sensitive to the human’s attentional state when producing facial expressions, suggesting that facial expressions are not just inflexible and involuntary displays of emotional states, but rather potentially active attempts to communicate with others.

## Introduction

Most mammalian species produce facial expressions, which form meaningful and adaptive components of the animal’s behavioural repertoire. The facial architecture underlying such facial expressions is highly conserved among mammals^1^, suggesting that human facial expression is based on evolutionarily ancient systems. Therefore, it would seem reasonable to debate the extent to which such facial expressions are underpinned by sophisticated cognitive processes. Historically, animal facial expressions (including human, to an extent) have been considered inflexible and involuntary displays e.g.^2,3^, reflecting an individual’s emotional state rather than active attempts to communicate with others. There is some evidence that non-human primate facial expressions can be mediated by the presence of an audience, suggesting that the sender has some understanding of whether the expressions can be seen by others^4–8^. Waller *et al*. (2015) showed that the production of facial expressions in orangutans is more intense and more complex during play when a recipient is directed towards them suggesting that the production of these expressions is not necessarily an automated response and subject to audience effects^6^. Similarly, Scheider *et al*. (2016) showed that Gibbons presented their facial expressions more often and over a longer duration when facing other individuals compared to non-facing situation^7^.

To date there is no systematic experimental evidence, however, that facial expressions in species other than primates, are produced with similar sensitivity to the attention of the audience.

With the current study, we aimed to test whether domestic dog facial expressions change in response to an highly arousing but non-social stimulus (food) and/or the changing attentional state of their human audience. Domestic dogs are a potentially interesting model for this kind of research as they have a unique history. Dogs have been living with humans for about 30,000 years^9^, during which time selection pressures seem to have acted on dogs’ ability to communicate with humans. [see for a review^10^ and^11,12^ for a recent discussion].

There is broad evidence that domestic dogs attend to a human’s attentional state^13–15^, which is one indicator of intentionality^16^. After being told not to take a piece of food, dogs steal the food more often when the human’s eyes are closed compared to situations during which the human’s eyes are open, the human has her back turned to the dog or she is distracted^13,15^. Dogs are also sensitive to the human’s attentional state during communicative interactions with humans. Dogs follow communicative gestures more once the humans eyes are visible and the gesture is clearly directed at them^16^. Dogs also follow the gaze of a human to a target only if eye contact had been established prior to the gaze shift^17^.

Waller *et al*. (2013) analysed the facial expressions of dogs waiting to be rehomed in shelters, and found a negative correlation between the frequency of facial movements the dogs produced when interacting with a stranger, and the rate at which they were re-homed. The more often dogs produced a specific facial movement, Action Unit 101 (which raises the inner eyebrow) the quicker they were re-homed^18^. Raising the inner eyebrow changes the visual appearance of the eyes and makes them look bigger, a key feature of paedomorphism (juvenile features present in the adult). One hypothesis is therefore that by picking dogs that raise their inner eye brow more, humans simply follow their preference for paedomorphic facial characteristics, a preference which might have acted as a selection pressure during dog domestication^18,19^.

It is possible, therefore, that dogs have also evolved the ability to use these facial expressions differentially depending on their audience. In which case, during domestication dogs may have gained additional cognitive control of their facial expressions.

The current study investigated whether dog facial expressions can be subject to so called audience effects, and can therefore be tailored to the human’s attentional state, which might suggest some social communicative function and possible voluntary control. The alternative is that dog facial expressions are a simple emotional display based on the dog’s state of arousal. In order to try and discriminate between these two possible explanations, the human, depending on the condition, presented a piece of food, as a non-social and arousing stimulus (a recent study, using thermal imaging, shows that food seems to be more arousing for dogs than social contact with a human as long as the human remains silent^20^).

Therefore, if dogs produce facial expressions merely as an emotional display, we would expect them to not necessarily differentiate between the social (human attention) and the non social (food) conditions. However, if the dogs behave in different ways in responds to the social and the non-social stimuli, this would provide some evidence that dogs discriminate between the conditions based on social context and one possible explanation for such discrimination that dogs exercise some voluntary control.

## Results

### Analysis of FACS Coding

A 2 × 2 × 2 multivariate MANOVA was conducted as the design used repeated measures and multiple dependent variables. There were three repeated measures: attention (attentive vs. not attentive), food (food present vs. food absent) and trial (trial 1 vs. trial 2). The multiple dependent variables were the nine AUs (see Table 1 for a list of AUs used in the analysis). To reduce the data and focus on the most commonly occurring movements, Action Units that one third of the dogs or more never produced in any of the four conditions were excluded from the analysis. The analysis showed no 3 - way interaction of attention, food and trial (Wilks’ λ = 0.058, *F*_(8,16)_, *p* = 0.24, η_*p*_^2^ = 0.43) and so we excluded the factor trial from further analysis.

**Table 1:** AU unit activity as a function of attention or not for individual univariate ANOVAs

AU/AD	*F(df)*	*p*	*d*	η_*p*_^2^	M(SD) *Attention*	M(SD) *No Attention*
**101**	**102.58 (1**,**23)**	**<**0.**0001**	**1.62**	0**.82**	0**.121 (**0**.04)**	0**.056 (**0**.04)**
145	0.04 (1,23)	=0.84	0.02	0.002	0.197 (0.06)	0.199 (0.09)
12	0.89 (1,23)	=0.35	0.09	0.04	0.044 (0.05)	0.039 (0.06)
25	1.33 (1,23)	=0.26	0.17	0.06	0.128 (0.12)	0.108 (0.11)
26	1.98 (1,23)	=0.17	0.21	0.08	0.139 (0.13)	0.113 (0.12)
118	0.88 (1,23)	=036	0.18	0.04	0.049 (0.07)	0.037 (0.05)
**19**	**5.87 (1,23)**	=0**.024**	**0.19**	0**.20**	0**.071 (**0**.07)**	0**.057 (**0**.07)**
102	2.21 (1,23)	=0.15	0.19	0.09	0.014 (0.02)	0.011 (0.01)
105	2.76 (1,23)	=0.11	0.26	0.11	0.047 (0.05)	0.036 (0.03)

Significant results in bold.

A 2 × 2 doubly multivariate MANOVA showed that there was no main effect of food, Wilks’ λ = 0.072, *F*_(9,15)_ = 0.65, *p* = 0.74, η_*p*_^2^ = 0.28 and no attention x food interaction, Wilks’ λ = 0.63, *F*_(9,15)_ = 95, *p* = 0.51, η_*p*_^2^ = 0.36. There was a significant main effect of attention with a large effect size, Wilks’ λ = 0.071, *F*_(9,15)_ = 21.78, *p* < 0.0001, η_*p*_^2^ = 0.93. The origin of the significant main effect of attention was that for all except one of the AUs (AU 145: blink), there was more activity in the attention condition than in the no attention condition (see Table 1).

**Figure 1:**
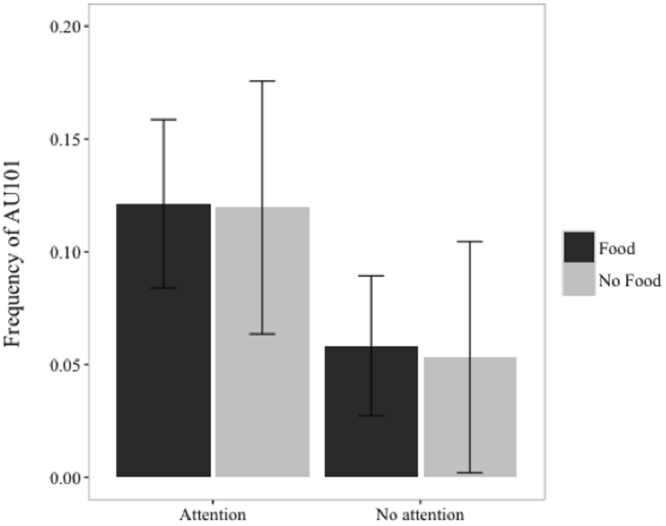
Mean rate (±1 SD) of facial movement AU101 (Inner eye brow raise) as a function of condition.

Two of the AUs, AU 101 (“eye brow raiser”, see Fig. 1) and AD 19 (“tongue show”) reached significance individually with the main factor attention having an effect on the amount of AU101 and AD19 movements produced (see Table 1, Fig. 1).

### Analysis of Behavioural Coding

We also examined the other behavioural measures to see if condition had an effect. We compared the frequency of vocalizing bouts across the four conditions using a Friedman test (data were non normally distributed). Condition had an effect on the frequency of vocalizations produced with a medium effect size, χ^2^(3, *N* = 23) = 17.69, *p* = 0.001, *W* = 0.26. The pattern of medians revealed that the human’s attention increased the frequency of the vocalizations produced by the dogs (attention/food *Mdn* = 2.76; attention/no food *Mdn* = 2.65; no attention/food *Mdn* = 2.37; no attention/no food *Mdn* = 2.11). Wilcoxon pairwise comparisons revealed significant differences between attention/food vs. no attention/food, *Z*(23) = 2.31, *p* = 0.021; attention/no food vs. no attention/food, *Z*(23) = 2.65*p* = 0.008; and attention/no food vs. no attention/no food, *Z*(23) = 2.49, *p* = 0.013. No other combinations revealed significant effects.

We also compared the frequency of tail wagging across the four conditions. There was no effect of condition, χ^2^(3, *N* = 23) = 6.49, *p* = 0.09, although the pattern of medians was similar to that found for vocalizing (attention/food *Mdn* = 2.76; attention/ no food *Mdn* = 2.65; no attention/food *Mdn* = 2.37; no attention/no food *Mdn* = 2.2) with attention and food increasing the frequency of the behaviour. None of the pairwise comparisons was significant.

We conducted similar analyses on the other behaviours (laying, sitting, standing and moving, see Table 1) but no significant effects were revealed.

## Discussion

The study has two main findings. First, human attentional state affected the production of dogs’ facial expressions. Dogs produced significantly more facial expressions when the human was oriented towards them, than when the human had her back turned to the dog. This effect was strongest for AUs, AU101 (“inner brow raiser”) and AD19 (“tongue show”). Human attentional state also affected one of the dogs other behaviours, the frequency of vocalizations produced. The visibility of the food, however, did not affect dogs’ facial movements and there is also no conclusive evidence that it affected any of the dogs other behaviours. So, while dogs produce more facial expressions when the human is oriented towards them and in a position to communicate, the visibility of non-social but arousing stimulus (the food) did not alter their facial movements in the same way.

One interpretation of the dogs’ behaviour could be that dogs produce their facial expressions communicatively and that the dogs’ facial expressions are not just mediated by the individual’s emotional state. Therefore, dogs increase the frequency of their production dependent on the other individual’s attentional state but not in response to being presented with a non social but arousing stimulus (the food). However, our data show an increase of all facial movements when the human is attentive, but no evidence that dogs specifically modulate their facial movements depending on the attentional state of the human. Human attention had an effect on one of the dogs other behaviours, the frequency of the vocalizations produced, but not their non-communicative behaviours, such as sitting and standing. Therefore, it seems that there might be a specific communicative function of this sensitivity to human attention. There is substantial evidence supporting the importance of visibility of the human’s eyes for dogs during communicative interactions with humans. Teglas *et al*. (2012) showed that dogs do not follow the gaze of a human to a certain location unless eye contact had been established beforehand^17^. Kaminski *et al*. (2012) showed that dogs follow a human’s communicative gestures (e.g. pointing or gazing) but ignore a human’s actions which resembled the communicative gestures but are not intended to be communicative and during which the humans eyes were not directed at the dog^16^. Here we now add to this evidence by showing that the visibility of the human’s eyes might be important for dogs for the production of facial expressions. This might be evidence that dogs produce facial expressions as a flexible signal and that its production depends on the attentional state of the receiver of the signal. Without taking any physiological measures it is obviously impossible to say to what extent seeing the human’s and the human’s eyes is also arousing for the dog. But our study highlights that a non-social stimulus which has been proven to be arousing for dogs, does not have any effect on the production of their facial expressions. It is however possible impossible to say whether dogs behaviour in this and other studies is evidence for a flexible understanding of another individuals perspective, hence constituting a true understanding of another individual’s mental state, or is a rather hardwired or learnt response to seeing the face or the eyes of another individual (see for a discussion^21,22^).

Interestingly there are two Action Units that stand out from the overall analysis, the AD 19 (tongue show) and the AU101 (inner brow raiser). The tongue shows movement can potentially be associated with stress (e.g. nose lick behavior) but could also indicate panting behavior, which dogs use for heat regulation^23^. However, a facial expression ethogram based on systematic analysis using tools like DogFACS does not exists, which is why we can only speculate. Interestingly in the general dog literature a relaxed open mouth with tongue show is sometimes described as generally attentive, which would be an interpretation in line with the results we see here.

However, AU101 may be of greatest significance as the response of humans to this unit may have had the greatest influence on selection. Waller *et al*. (2013) showed that dogs from a shelter that produced the AU101 more frequently were rehomed quicker^18^. This could be for two possible reasons. Firstly, AU101 resembles a facial movement which in humans indicates sadness, hence potentially making humans feels more empathic towards dogs that produce this movement more. Another possibility is that the AU101 lets the eyes of the dogs appear bigger and more infant like potentially tapping into the preference of humans for paedomorphic characteristics and/ or humans innate tendency to respond to ostensive cues, one of which is ‘eyebrow raising’^24,25^. Regardless of the exact mechanism, it seems that humans are particularly responsive to this facial movement in dogs. Increased production of this movement in response to human attention could benefit dogs in their interaction with humans, therefore.

In conclusion, we have demonstrated that dogs’ production of facial expressions is subject to audience effects, and can be tailored to the human attentional state suggesting some communicative function and are not simple emotional displays based on the dogs arousal state. Facial expressions are often considered to be an automatic, reflexive and emotionally based system^3^, but these data point to a more flexible system (at least in domestic dogs) combining both emotional and potentially cognitive processes.

## Methods

### Subjects

24 family dogs (13 male and 11 female) of various breeds and ages (age range = 1–12 years, *M*age = 4.75, *SD* = 3.33) participated in the study (see Table 2). The dogs were normal family dogs with a training background typical for a pet dog. Dogs were randomly selected from a database of dogs at the Max Planck Institute for Evolutionary Anthropology in Leipzig/ Germany. The only criterion for selection was that dogs had to be comfortable to be without their owner and comfortable with a stranger in a strange environment. Research was non-invasive and strictly adhered to the legal requirements of Germany. The study was ethically approved by an internal committee at the Max Planck Institute for Evolutionary Anthropology (members of the committee are Prof. M. Tomasello, Dr. J. Call and Susanne Mauritz). The animal research complies with the “Guidelines for the Treatment of Animals in Behavioral Research and Teaching” of the Association for the Study of Animal Behavior (ASAB). IRB approval was not necessary because no special permission for the use of animals in purely behavioural or observational studies is required in Germany (TierSchGes §7 and §8). Dogs were fed by their owners according to their normal daily routine and not food deprived in any way. Water was available to the dogs ad libitum. In the conditions during which food was presented to the dogs, ©Frolic was used, which is a food mainly used by owners as a treat in between meals. Dogs received regular breaks and observations were stopped in case the dogs showed any sign of severe stress.

**Table 2:** List of the dogs included in the study with information about breed, gender and age (years).

Name	Breed	Gender	Age (Years)
Anouk	Eurasier	Male	1
Arik	Hovawart	Male	4
Bacardi	Mongrel (German Shepherd mix)	Female	11
Balou	Schapendoes	Male	11
Basma	Basenji	Female	2
Caja	Mongrel (Doberman Mix)	Female	8
Cody	Mongrel	Male	4
Dusky	Basenji	Male	2
Fefo	Parson Jack Russell	Male	3
Gerda	Mongrel (Poodle & Labrador Mix)	Female	3
Gordo	Mongrel (Canario & Doberman Mix)	Male	4
Guenni	Whippet	Male	2
Guiness	Dalmation	Male	5
Kendra	Border Collie	Female	2
Kenny	Labrador	Male	3
Lea	Mongrel (Leonberger & German Shepherd Mix)	Female	11
Luna	German Shepherd	Female	4
Mira	Mongrel (Podenco & Magyar Vizsla Mix)	Female	5
Paul	Golden Retriever	Male	3
Romy	Mongrel (Rottweiler Mix)	Male	2
Scully	Border Collie	Female	5
Sparky	Boxer	Male	2
Tina	Mongrel	Female	12
Wilma	Mongrel (Rottweiler and Rhodesian Ridgeback Mix)	Female	5

### Materials

**Figure 2:**
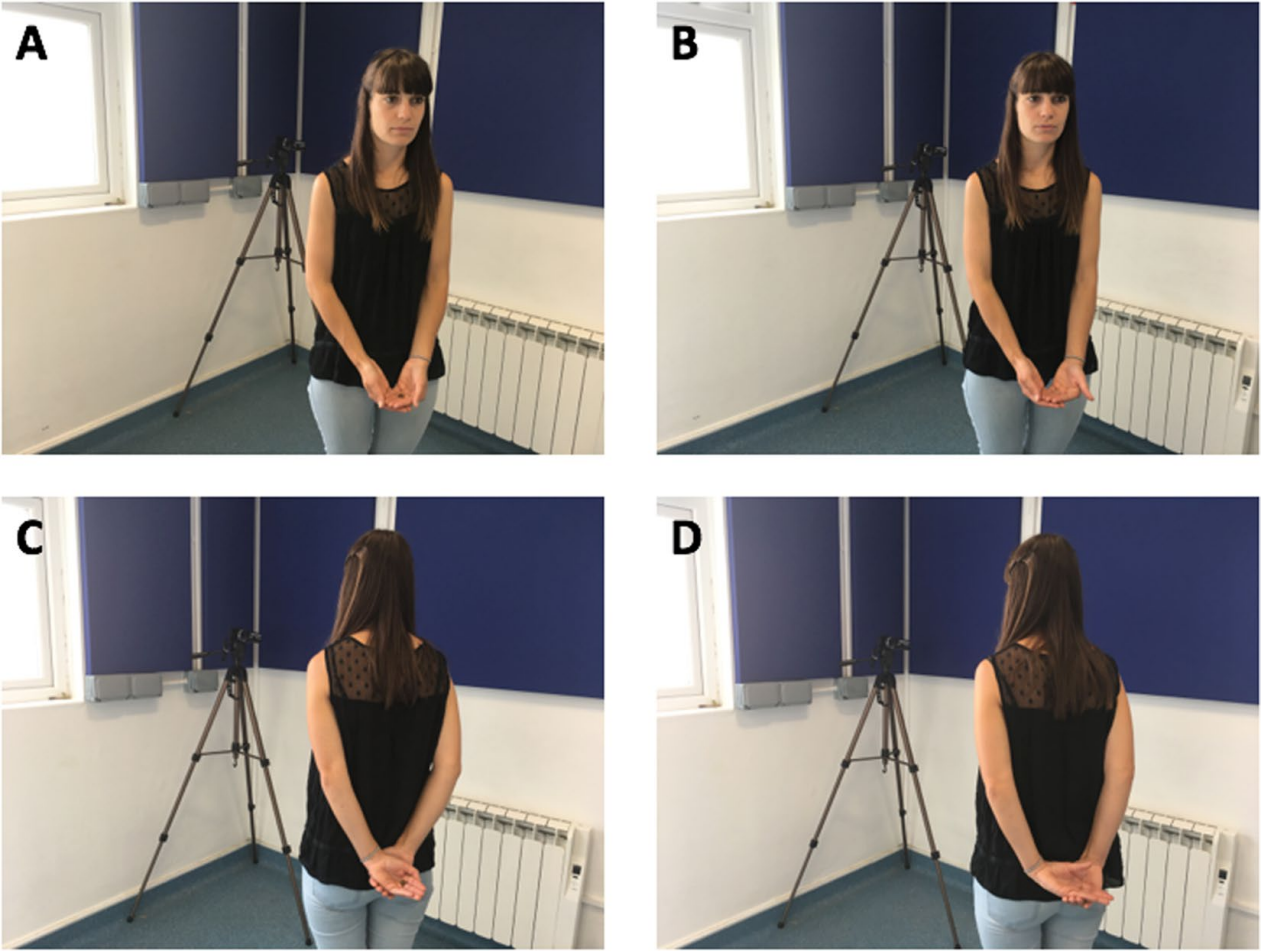
Experimenter’s position in the (**A**) Attentive Food (**B**) Attentive No food (**C**) Not attentive Food (**D**) Not attentive no Food condition.

Dogs were observed in a quiet room (2.85 m × 3.60 m) and to ensure that dogs did not move around extensively so their facial expressions could be observed easily, dogs were tied with a lead (1 m) to a predetermined spot in the room. Dogs were situated 1 m away from the human who was standing on a predetermined and marked spot. A video camera was placed on a stationary tripod such that the dogs’ faces were visible (see Fig. 2).

### Procedure

Before testing started, each dog was allowed to familiarise itself with the experimenter and the room in which testing was conducted, and was allowed to move around freely for several minutes. After that the dog was attached to a lead and the experimenter positioned herself in front of the dog and behaved according to the following four conditions:

#### Attentive Food

The experimenter stood facing the dog with arms pointing towards the dog and palms placed together displaying the food (see Fig. 2A).

#### Attentive No Food

The experimenter stood facing the dog with palms in the same position but not displaying food (see Fig. 2B).

#### Not Attentive Food

The experimenter had her back turned towards the dog but had her arms behind her back, palms placed together displaying the food (see Fig. 2C).

#### Not Attentive No Food

The experimenter had her back turned towards the dog and her arms behind her back, palms placed together but not displaying food (see Fig. 2D).

The design was a within subjects design in which each dog received all four conditions. The order of conditions was counterbalanced across dogs.

During each trial the experimenter stood still and did not respond to any of the dog’s behaviours. The experimenter looked at a predetermined spot at the opposite wall and did not actively seek eye contact with the dog when she was oriented towards the dog. After 2 minutes the trial ended and the human briefly interacted with the dog before she then changed her position according to the condition presented in the next trial.

Each dog received two trials per condition, summing up to 8 trials altogether. Trials were split in two sessions to prevent fatigue and sessions were presented on two different days, with a break of up to four days between sessions. The order of condition was counterbalanced across dogs.

### FACS Coding

Coding of the facial movements was based on the DogFACS manual (Waller *et al*. 2013: www.dogfacs.com). DogFACS (Waller *et al*., 2013) is based on The Facial Action Coding System (FACS), an anatomically based facial expression coding system first developed for humans (Ekman & Friesen, 1978). It identifies observable facial changes associated with underlying muscle movement (Action units, AUs) allowing an objective, reliable and standardized measurement of facial movements. DogFACS was used to identify the facial movements produced during each condition. A certified DogFACS coder (JH) coded the frequency and duration of the different action units. Action Units that one third of the dogs or more never produced in any of the four conditions were excluded from the analysis (for a list of all possible AUs, see Waller *et al*. 2013 or www.dogfacs.com). This left 9 AUs (Action Units), ADs (Action Descriptors) and EADs (Ear Action Descriptors) that were included in the analysis (see Table 3 for a description).

**Table 3:** Facial movements (DogFACS: Action Units, AUs, Action Descriptors, ADs and Ear Action Descriptors, EADs) and general behaviours coded.

Behaviour	Definition
AU 101^(1)^	Inner brow raiser. Lifting of the inner brow region performed by the frontalis muscle.
AU 145^(1)^	Blink: the relaxation of the levator palpebrae muscle and contraction of the orbicularis occuli circular muscle act to move the upper and lower eyelid, closing the eye. The retractor anguli occuli lateralis pulls the outer corner of the eye caudally, aiding in closing the eye.
AU 12^(1)^	Lip corner puller. The zygomaticus pulling the lip corners towards the ears.
AU 25^(1)^	Lips part.
AU 26^(1)^	Jaw drop.
AU 118^(1)^	Lip pucker. The buccinators and orbicularis oris muscles act to push the lip corners rostrally, towards a medial point.
AD 19^(1)^	Tongue show. The tongue is shown and it reaches at least the inner lower lip.
EAD 102^(1)^	Ears adductor. The ears are adducted and the base of both pinnas becomes closer together by being pulled towards the head midline.
EAD 105^(1)^	Ears downward. The ears are pulled ventrally, laterally.
Laying^(2)^	The dog’s legs laid flat on the ground while the head could rest on the ground, legs, or remain off the ground.
Sitting^(2)^	The dog’s forelegs were extended and perpendicular to the ground while the hind legs were flexed with the tarsus resting flat on the ground.
Standing^(2)^	All legs were extended and perpendicular to the ground.
Moving^(2)^	The dog displaced its body from one location to another by alternately moving its legs more than two steps.
Tail-wagging	The dog’s tail is extended and moves side to side in quick motion.
Vocalising	Any sound emitted from the dog.

Listed definitions were partly obtained from ^(1)^Waller *et al*. 2013 and ^(2)^Call *et al*. (2003).

### Behavioural Coding

In addition to the FACS coding we also coded the general behaviour of the dogs to see if we could identify any changes across conditions. We coded lying, sitting, standing, moving, tail-wagging, yawning, nose licking and vocalizing (see Table 3 for a list of the coded behaviours and their definitions).

### Reliability coding

For the FACS coding JH (a trained FACS coder) did intra-rater reliability coding of 20% of the original material (frequency of AUs produced) 1 year after her original coding. Reliability was excellent with all r_ic_ > 0.88, N = 40, *p* < 0.001. For the behavioural data (frequency of behaviours), a second coder unaware of the research question coded 20% of the original material for the different behaviours. Reliability was excellent for each of the behaviours (Stand: r_ic_ = 0.099, N = 40, *p* < 0.0001, Sit: r_ic_ = 0.99, N = 40, *p* < 0.0001 Move: r = 0.98, N = 40, *p* < 0.0001, Lying: r_ic_ = 1, N = 40, *p* < 0.0001, Tail wagging: r_ic_ = 0.99, N = 40, *p* < 0.0001Vocalize: r = 0.99, N = 40, *p* < 0.0001).

### Data Availability Statement

All data will be made available.
